# Determining association between blood glucose variability and postoperative delirium in acute aortic dissection patients: methodological issues

**DOI:** 10.1186/s13019-021-01715-4

**Published:** 2021-12-04

**Authors:** Cheng-Wen Li, Fu-Shan Xue, Bin Hu

**Affiliations:** Department of Anesthesiology, Beijing Friendship Hospital, Capital Medical University, NO. 95 Yong-An Road, Xi-Cheng District, Beijing, 100050 People’s Republic of China

**Keywords:** Glucose variability, Postoperative delirium, Risk factors, Prediction, Acute aortic dissection

## Abstract

The letter to the editor made several comments regarding possible methodological issues in the recent article by Lin et al. determining the association between blood glucose variability and postoperative delirium in patients undergoing acute aortic dissection surgery with cardiopulmonary bypass, which is published in ***Journal of Cardiothoracic Surgery.*** 2021; 16(1):82. Our concerns included the lack of some important perioperative factors associated with postoperative delirium, the process of establishing multivariate model and the method of using the receiver operating characteristic curve analysis to assess the predictive performance of the standard deviation of blood glucose for the development of POD. We would like to invite the authors to comment on these and believe that clarifying these issues would improve the transparency of this study and interpretation of findings.

***Re***: ***Lin YJ, et al.*** Association between glucose variability and postoperative delirium in acute aortic dissection patients: an observational study. J Cardiothorac Surg. 2021; 16(1):82.

**To the Editor**

We have a great interest in the recent article by Lin et al.[1] determining the association between blood glucose variability and postoperative delirium (POD) in patients undergoing acute aortic dissection surgery with cardiopulmonary bypass (CPB). By the multivariate Cox regression analysis, they showed that blood glucose variability was significantly associated with the risk of POD. Given that POD is one of most common complications after cardiovascular surgery with CPB and has been significantly associated with increased length of hospital stay, costs, morbidity and mortality in patients undergoing acute aortic dissection surgery with CPB [2], their findings have potentially clinical implications. However, we noted several issues in their methodology on which we would like to invite the authors to comment.

First, the authors did not perform the preoperative neuropsychiatric assessment of patients. The available literature indicates that preoperative anxiety, depression and decreased mini-mental state exam score are the independent risk factors of POD after cardiovascular surgery with CPB [3, 4]. Furthermore, the readers were not provided with the details of anesthetic and perioperative managements. Thus, it is difficult to estimate the extent of influence that anesthetic and intraoperative interventions might have on the development of POD. Other than operating time, CPB time, aortic cross-clamp time and blood loss described in this study, it has been shown that an increased dose of fentanyl, antegrade selective cerebral perfusion time, lowest hemoglobin level, lowest mean arterial pressure, lowest body temperature and partial pressure of oxygen during CPB and intraoperative blood transfusions can significantly increase the risk of POD following cardiac and aortic dissection operations [3, 5–7]. In a retrospective or observational study, multivariate analysis indeed is a useful statistical method for determining risk factors of adverse perioperative events by adjusting patients’ demographic data and controlling selection biases. To obtain the true inferences of multivariate analysis for adjusted hazard ratio of measured outcome, however, it is generally believed that all of known factors affecting measured outcome must be taken into the account. If an important risk factor is missed, multivariate adjustment for hazard ratio of measured outcome can be biased and even a spurious association between intervention and outcome of interest may be obtained [8]. Thus, we argue that not taking above preoperative and intraoperative factors associated with the risk of POD into the account would have distorted the inferences of the multivariate Cox regression analysis and biased the adjusted hazard ratios of interested variables including blood glucose variability in this study.

Second, the authors did not provide the details of building two multivariate Cox regression models for identification of possible predictors for the development of POD. Thus, it was unclear why the authors only selected age, male, first time blood glucose level, neutrophil and white blood cell counts, hypoxemia, mechanical ventilation duration, Acute Physiology and Chronic Health Evaluation Score of more than 20, and standard deviation of blood glucose as covariate variables to be included into the multivariate model for statistical adjustment. Generally speaking, when building a multivariate model, all candidate covariate variables must be entered into the univariate model to examine multicollinearity. Then, the covariate variables with large *P* values (P < 0.2) in the univariate analyses can be included into the multivariate model using POD as the dependent outcome variable for identification of independent risk factors for the occurrence POD, with their *P* values, adjusted odds ratios and 95% confidence intervals [8]. As an important step of building the multivariate model may have been ignored in this study, we are concerned that their results of multivariate analyses determining the association between blood glucose variability and POD would have been biased due to multicollinearity.

Finally, the receiver operating characteristic (ROC) curve analysis was used to assess predictive performance of the standard deviation of blood glucose for the development of POD. After the ROC curve analysis, the authors provided its area under the ROC curve, sensitivity and specificity, but not its Youden index at the optimal cutoff point, positive and negative predictive values. Thus, the readers cannot determine whether blood glucose variability really has a good predictive ability for the occurrence of POD, despite it has an areas under the ROC curve of 0.763 [9].

## Data Availability

Not applicable.

## Abbreviations

POD: Postoperative delirium
CPB: Cardiopulmonary bypass
ROC: Receiver operating characteristic curve
